# Eriocitrin Disrupts Erythrocyte Membrane Asymmetry through Oxidative Stress and Calcium Signaling and the Activation of Casein Kinase 1α and Rac1 GTPase

**DOI:** 10.3390/ph16121681

**Published:** 2023-12-02

**Authors:** Sumiah A. Alghareeb, Jawaher Alsughayyir, Mohammad A. Alfhili

**Affiliations:** Department of Clinical Laboratory Sciences, College of Applied Medical Sciences, King Saud University, Riyadh 12372, Saudi Arabia; 442204700@student.ksu.edu.sa (S.A.A.);

**Keywords:** eriocitrin, hemolysis, eryptosis, calcium, oxidative stress

## Abstract

Background: Hemolysis and eryptosis result in the premature elimination of circulating erythrocytes and thus contribute to chemotherapy-related anemia, which is extremely prevalent in cancer patients. Eriocitrin (ERN), a flavanone glycoside in citrus fruits, has shown great promise as an anticancer agent, but the potential toxicity of ERN to human erythrocytes remains unstudied. Methods: Erythrocytes were exposed to anticancer concentrations of ERN (10–100 μM) for 24 h at 37 °C, and hemolysis and associated markers were quantified using colorimetric assays. Eryptosis was assessed by flow cytometric analysis to detect phosphatidylserine (PS) exposure by annexin-V-FITC, intracellular Ca^2+^ using Fluo4/AM, and oxidative stress with 2-,7-dichlorodihydrofluorescin diacetate (H_2_DCFDA). ERN was also tested against specific signaling inhibitors and anti-hemolytic agents. Results: ERN caused significant, concentration-dependent hemolysis at 20–100 μM. ERN also significantly increased the percentage of eryptotic cells characterized by Ca^2+^ elevation and oxidative stress. Furthermore, the hemolytic activity of ERN was significantly ameliorated in the presence of D4476, NSC23766, isosmotic urea and sucrose, and polyethylene glycol 8000 (PEG). In whole blood, ERN significantly elevated MCV and ESR, with no appreciable effects on other peripheral blood cells. Conclusions: ERN promotes premature erythrocyte death through hemolysis and eryptosis characterized by PS externalization, Ca^2+^ accumulation, membrane blebbing, loss of cellular volume, and oxidative stress. These toxic effects, mediated through casein kinase 1α and Rac1 GTPase, can be ameliorated by urea, sucrose, and PEG. Altogether, these novel findings are relevant to the further development of ERN as an anticancer therapeutic.

## 1. Introduction

Flavonoids, glycosides, carotenoids, alkaloids, and terpenes comprise the most significant categories of metabolites in medicinal plants and *Citrus* fruits [1,2]. Collectively, these active metabolites are responsible for the wide spectrum of pharmacological properties attributed to *Citrus* fruits, including antioxidant, anti-inflammatory, antidiabetic, antimicrobial, anticancer, neuroprotective, and cardioprotective activities [1,3,4]. In particular, a large number of studies have concluded that flavonoids improve the symptoms of a plethora of pathological conditions, including diabetes mellitus [5], neurodegenerative disease [6], and respiratory disease, among others [7]. Furthermore, flavonoids improve cognitive function in humans [8] and favorably influence gastrointestinal inflammation by interacting with the gut microbiome [9].

Eriocitrin (ERN) is a flavanone-*7*-*O*-glycoside with antioxidant, anti-inflammatory, anticancer, and anti-allergic properties [9]. When compared to other antioxidants, ERN was more effective in scavenging free radicals associated with diabetes mellitus and other chronic illnesses [10,11]. Notably, several studies have shown that ERN stimulates apoptosis in nucleated cells. In HL-60 cells, ERN causes caspase-dependent apoptosis characterized by DNA fragmentation and chromatin condensation [12]. ERN also inhibits the proliferation of HepG2 and Huh7 liver cells through the intrinsic apoptosis pathway and cell cycle arrest [13]. Moreover, ERN promotes intrinsic apoptosis in MCF-7 cells through JAK2/STAT3/Src inhibition and reactive oxygen species (ROS)-dependent JNK/p38 MAPK stimulation [14]. Very recently, ERN has been reported to trigger ferroptosis in A549 and H1299 lung adenocarcinoma cells [15], thereby circumventing cancer cell resistance to apoptosis. Altogether, mounting studies are arguing for the further development of ERN as an anticancer therapeutic.

The prevalence of chemotherapy-induced anemia (CIA) is at least 75% in cancer patients undergoing treatment [16]. Although poorly understood, CIA may be caused by bone marrow suppression, hemolysis, and eryptosis [17]. Eryptotic erythrocytes lose the asymmetrical arrangement of the phospholipids on their cell membrane, culminating in phosphatidylserine (PS) externalization. PS, in turn, serves as a binding site for phagocytes to eliminate aged, infected, and damaged cells from the circulation which prevents intravascular hemolysis. However, premature eryptosis, as instigated by chemotherapeutic drugs, leads to the excessive elimination of circulating RBCs, which gives rise to anemia [17]. Other hallmarks of eryptotic cells include dehydration and shrinkage, cytosolic calcium buildup, oxidative stress, ceramide accumulation, and metabolic exhaustion [18]. The signaling mediators involved in eryptosis include caspase, p38 MAPK, casein kinase 1α (CK1 α), protein kinase C, and Rac1 GTPase, among others [18,19].

Numerous chemotherapeutic drugs are known to cause anemia in patients; most notably paclitaxel and cisplatin, have been found to induce eryptosis [20,21]. However, the hemolytic and eryptotic potential of ERN against human RBCs remains unknown. The aim of the present study was to examine the potential toxic effects of ERN on erythrocytes and the associated molecular mechanisms to optimize the further development and validation of the flavanone as an anticancer agent.

## 2. Results

### 2.1. ERN Induces Concentration-Dependent Hemolysis

**Figure 1:**
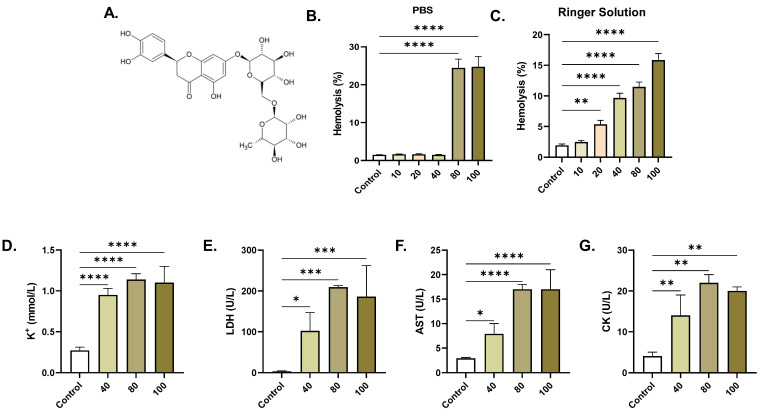
ERN induces hemolysis. (**A**) Molecular structure of ERN. (**B**) Concentration-responsive hemolytic activity of ERN (10–100 μM) in PBS. (**C**) Concentration-responsive hemolytic activity of ERN (10–100 μM) in Ringer solution. ERN-induced (40–100 μM) leakage of hemolytic markers (**D**) K^+^, (**E**) LDH, (**F**) AST, and (**G**) CK. Results are shown as means ± SEM (*n* = 9). ns indicates no statistical significance, while * (*p <* 0.05), ** (*p <* 0.01), *** (*p <* 0.001), and **** (*p <* 0.0001).

Relative to control values of 1.46% ± 0.09, the hemolytic rate of ERN in saline was significantly increased to 24.43% ± 2.31 (*p <* 0.0001) at 80 µM and to 24.73% ± 2.72 (*p <* 0.0001) at 100 µM, as seen in Figure 1B. In Ringer solution (Figure 1C), ERN elicited significant hemolysis, with increases in the control cells from 1.92% ± 0.68 to 5.35% ± 1.99 (*p <* 0.01, 20 μM), 9.65% ± 2.37 (*p <* 0.0001, 40 μM), 11.47% ± 2.35 (*p <* 0.0001, 80 μM), and 15.84% ± 3.22 (*p <* 0.0001). Accordingly, there was a significant release of K^+^ from 0.26 ± 0.03 to 0.97 ± 0.032 mmol/L, *p <* 0.0001 (40 µM), 1.15 ± 0.03 mmol/L, *p <* 0.0001 (80 µM), and 1.163 ± 0.068 mmol/L, *p <* 0.0001 (100 µM), as demonstrated in Figure 1D. LDH activity (Figure 1E) in the control supernatants (3.5 ± 0.72 U/L) similarly increased to 104.0 ± 24.27 U/L, *p* = 0.0168 (40 µM), 206.7 ± 4.49 U/L, *p* = 0.0002 (80 µM), and 203.0 ± 30.37 U/L, *p* = 0.0003 (100 µM). Furthermore, AST activity significantly increased from 2.5 ± 0.5 U/L in the supernatants of the control cells to 8.3 ± 0.86 U/L, *p <* 0.0001 (40 µM), 1.15 ± 0.03 U/L, *p <* 0.0001 (80 µM), and 1.163 ± 0.068 U/L, *p <* 0.0001 (100 µM), as shown in Figure 1F. CK activity (Figure 1G) followed a similar pattern, with a significant increase from 3.67 ± 0.88 (control) to 15.33 ± 1.86 U/L, *p* = 0.0083 (40 µM), 20.0 ± 3.06 U/L, *p* = 0.0011 (80 µM), and 19.0 ± 1.52 U/L, *p* = 0.0016 (100 µM).

### 2.2. ERN Increases Extracellular Acidity

**Figure 2:**
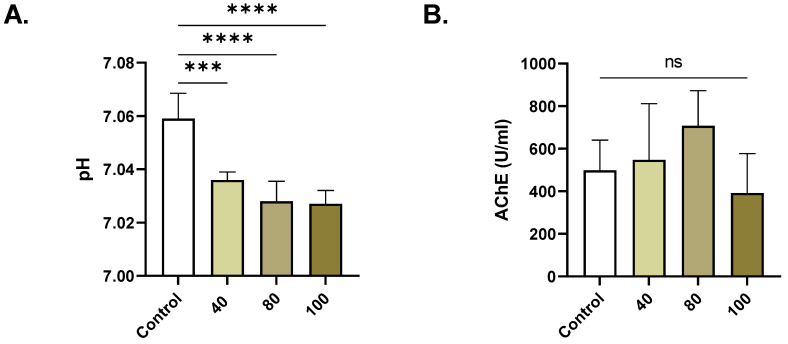
Effect of ERN on extracellular pH and AChE. (**A**) pH and (**B**) AChE activity in control and ERN-treated (40–100 μM) cells. Results are shown as means ± SEM (*n* = 9). ns indicates no statistical significance. *** (*p <* 0.001); **** (*p <* 0.0001).

ERN at 40, 80, and 100 µM significantly decreased the pH (Figure 2A) from 7.06 ± 0.003 to 7.031% ± 0.004 (*p* = 0.0002), 7.024 ± 0.004 (*p <* 0.0001), and 7.021% ± 0.006 (*p <* 0.0001), respectively. No significant anticholinesterase activity was observed for ERN (Figure 2B).

### 2.3. ERN Stimulates Eryptosis

**Figure 3:**
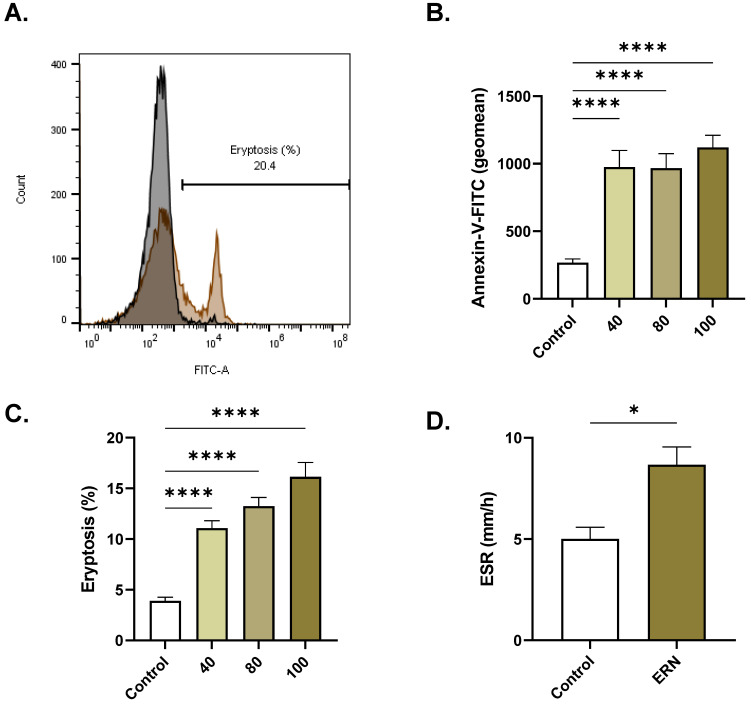
ERN induces eryptosis. (**A**) Representative histograms of annexin-V-FITC fluorescence of control (black line) and treated cells (100 μM; brown line). (**B**) Geomean annexin-V-FITC fluorescence (a.u.) of control and treated (40–100 μM) cells. (**C**) Percentage of eryptotic cells (40–100 μM). (**D**) ESR of control and treated (100 μM) cells. Results are shown as means ± SEM (*n* = 9). * (*p <* 0.05) and **** (*p <* 0.0001).

In Figure 3B, the geometric mean of annexin-V-FITC fluorescence was significantly elevated from 267.7 ± 27.37 a.u. (control) to 976.2 ± 121.8 a.u. (40 µM, *p* < 0.0001), 967.2 ± 107.2 a.u. (80 µM, *p* < 0.0001), and 112.1 ± 89.51 a.u. (100 µM, *p* < 0.0001), as was the percentage of eryptotic cells (Figure 3C). The ESR (Figure 3D) of the ERN-treated cells (100 µM) showed a significant increase compared to that of the control cells (5.0 ± 0.58 mm/h to 8.67 ± 0.88 mm/h, *p* = 0.0254).

### 2.4. ERN Elevates Cytosolic Ca^2+^

**Figure 4:**
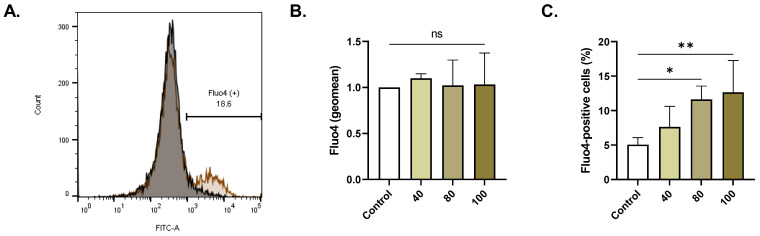
ERN raises cytosolic Ca^2+^ levels. (**A**) Representative histograms of Fluo4 fluorescence of control (black line) and treated cells (100 μM; brown line). (**B**) Geomean Fluo4 fluorescence (fold change) of control and treated (40–100 μM) cells. (**C**) Percentage of cells with excess Ca^2+^ accumulation (40–100 μM). Results are shown as means ± SEM (*n* = 9). ns indicates no statistical significance, while * (*p <* 0.05) and ** (*p <* 0.01).

As depicted in Figure 4C, the percentage of cells with increased intracellular Ca^2+^ significantly increased to 10.79% ± 1.28 (*p <* 0.05, 80 µM) and 12.62% ± 2.12 (*p <* 0.01, 100 µM) compared to a control value of 5.03% ± 0.58.

### 2.5. ERN Elicits Oxidative Stress

**Figure 5:**
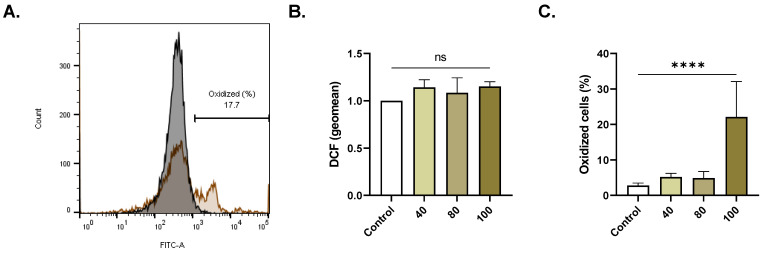
ERN elicits oxidative damage. (**A**) Representative histograms of DCF fluorescence of control (black line) and treated cells (100 μM; brown line). (**B**) Geomean DCF fluorescence (fold change) of control and treated (40–100 μM) cells. (**C**) Percentage of oxidized cells (40–100 μM). Results are shown as means ± SEM (*n* = 9). ns indicates no statistical significance, while **** (*p <* 0.0001).

Figure 5C illustrates that exposure to 100 µM of ERN caused a significant increase in the proportion of cells with intracellular ROS (2.76% ± 0.33 to 21.0% ± 6.11, *p <* 0.0001).

### 2.6. ERN Causes Cell Shrinkage and Swelling

**Figure 6:**
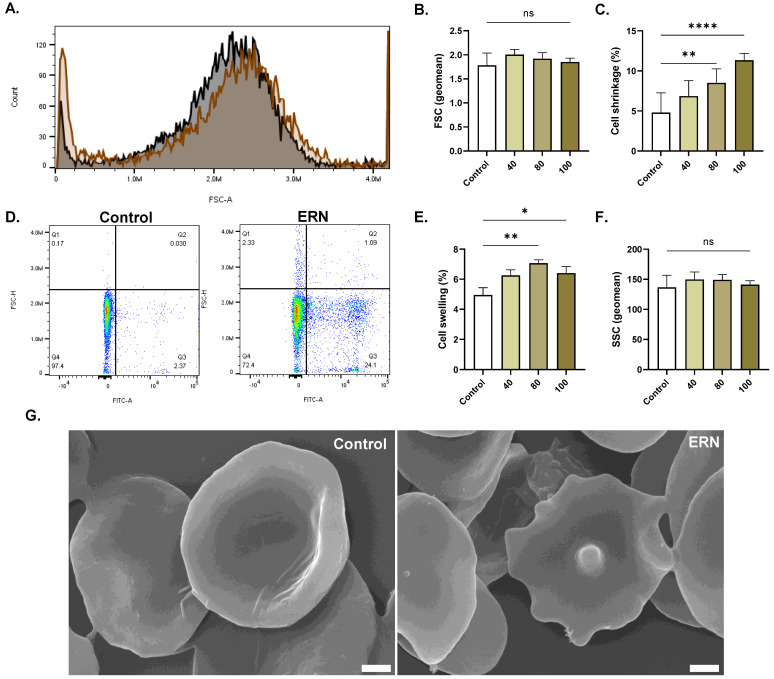
Effect of ERN on RBC morphology. (**A**) Representative histograms of FSC of control (black line) and treated cells (100 μM; brown line). (**B**) Geomean FSC (a.u.) of control and treated (40–100 μM) cells. (**C**) Percentage of shrinkage in control and treated (40–100 μM) cells. (**D**) Distribution of control and treated (100 μM) cells relative to FSC-H and annexin-V-FITC. (**E**) Percentage of swelling in control and treated cells (40–100 μM). (**F**) Geomean SSC (a.u.) of control and treated (40–100 μM) cells (**G**) SEM micrographs (×10,000; scale bar: 1 μM) of control and treated (100 μM) cells. Results are shown as means ± SEM (*n* = 9). ns indicates no statistical significance, while * (*p <* 0.05), ** (*p <* 0.01), and **** (*p <* 0.0001).

The percentage of cells undergoing shrinkage (Figure 6C) was significantly increased from 5.55% ± 0.49 in the control group to 8.80% ± 0.79 (*p* = 0.0081) and 11.33% ± 0.85 (*p <* 0.0001) after treatment with 80 µM and 100 µM of ERN, respectively. Moreover, the proportion of swollen cells (Figure 6E) also significantly increased from 4.95% ± 0.49 to 7.06% ± 0.23 (80 µM, *p* = 0.0016) and to 6.41% ± 0.43 (100 µM, *p* = 0.033).

### 2.7. CK1α and Rac1 GTPase Mediate ERN-Induced Hemolysis

**Figure 7:**
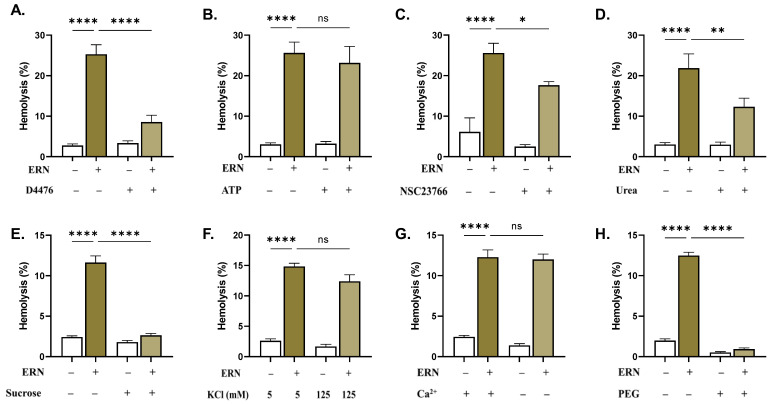
Inhibitors of ERN-induced RBC death. Effect of (**A**) D4476 (20 uM), (**B**) ATP (500 μM), (**C**) NSC23766 (100 uM), (**D**) urea (300 mM), (**E**) sucrose (250 mM), (**F**) KCl (125 mM), (**G**) extracellular Ca^2+^ (1 mM) removal, and (**H**) PEG 8000 (10%) on ERN-induced hemolysis (80 μM). Results are shown as means ± SEM (*n* = 9). ns indicates no statistical significance, while * (*p <* 0.05), ** (*p <* 0.01), and **** (*p <* 0.0001).

Compared to those treated with ERN, the cells exposed to a combination of ERN (80 μM) and D4476 exhibited significantly reduced hemolysis (25.28% ± 2.36 to 8.56% ± 1.68, *p <* 0.0001, Figure 7A). Likewise, cells were rescued from hemolysis in the presence of NSC23766 (25.58% ± 2.41 to 17.63% ± 0.89, *p* = 0.0376, Figure 7C). Additionally, hemolysis was significantly ameliorated by isosmotic urea (21.85% ± 3.51 to 12.3% ± 2.15, *p* = 0.0079) and sucrose (11.63% ± 0.82 to 2.62% ± 0.25, *p <* 0.0001), as revealed in in Figure 7D and 7E, respectively. Nevertheless, a complete inhibition of hemolysis was only seen with PEG 8000 (12.48% ± 0.41 to 0.94% ± 0.14, *p <* 0.0001, Figure 7H).

### 2.8. ERN Exhausts Intracellular Hb Stores

**Figure 8:**
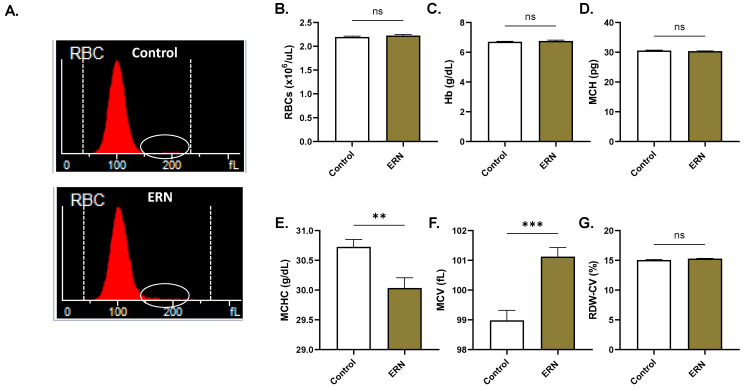
ERN exhausts corpuscular Hb in whole blood. (**A**) Representative histograms of RBC volume. (**B**) RBC count. (**C**) Hb. (**D**) MCH. (**E**) MCHC. (**F**) MCV. (**G**) RDW-CV. Results are shown as means ± SEM (*n* = 9) for control and treated (100 μM) whole blood. ns indicates no statistical significance. ** (*p <* 0.01); *** (*p <* 0.001).

**Figure 9:**
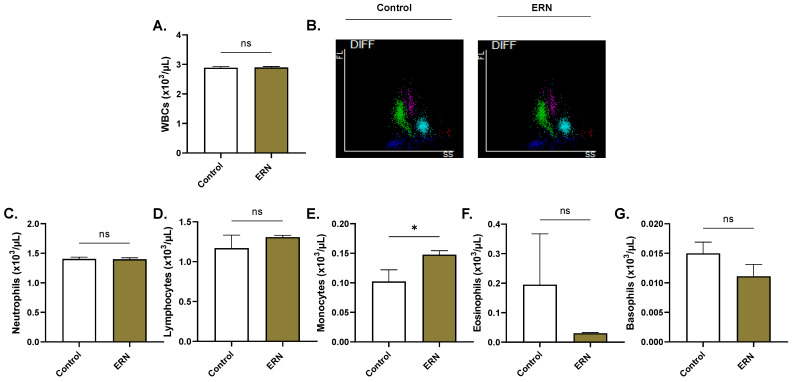
Effect of ERN on white blood cells. (**A**) Leukocyte count. (**B**) Representative scattergrams of fluorescence and side scatter intensity of leukocytes. Viability of (**C**) neutrophils, (**D**) lymphocytes, (**E**) monocytes, (**F**) eosinophils, and (**G**) basophils. Results are shown as means ± SEM (*n* = 9) for control and treated (100 μM) whole blood. ns indicates no statistical significance, while * (*p <* 0.05).

In whole blood, MCHC was significantly lower compared to the control samples (30.73 ± 0.13 g/dL to 30.03 ± 0.17 g/dL, *p* = 0.0068), as illustrated in Figure 8E. Additionally, ERN treatment resulted in a statistically significant increase in MCV (Figure 8F) from (from 98.98 ± 0.34 fL to 101.1 ± 0.31 fL, *p* = 0.0133) and monocytes (from 0.10 ± 0.02 × 10^3^/µL to 0.15 ± 0.01 × 10^3^/µL, *p* = 0.0453), as shown in Figure 9E.

## 3. Discussion

The current study reveals, for the first time, that anticancer concentrations of ERN (20–100 μM) [12,13,14,15] stimulate premature RBC death by both hemolysis and eryptosis. However, whether this concentration range is achievable in vivo remains to be determined in future studies. In this work, ERN was found to induce concentration-dependent hemolysis (Figure 1), indicating its detrimental effects on the integrity of the RBC membrane and the subsequent release of cellular contents including Hb, K^+^, LDH, AST, and CK. Extracellular Hb can potentially exacerbate inflammatory reactions and lead to excessive tissue damage and organ failure, as observed in hemolytic uremic syndrome [22,23]. Our results show that ERN significantly decreased extracellular pH (Figure 2), indicating the acidification of the cellular milieu, which appears to be associated with oxidative hemolysis (Figure 5) [24].

Loss of membrane asymmetry and PS externalization (Figure 3) flags cells for elimination from the bloodstream. Also, eryptotic cells lose membrane elasticity and adhere to endothelial cells which predisposes to thrombosis [18]—an observation further inferred from the high ESR in the treated cells (Figure 3). Eryptosis may be viewed as a defense mechanism that eliminates senescent and damaged RBCs to protect against hemolytic and thromboembolic events. However, inordinate or premature eryptosis, as demonstrated by ERN in this report, may exceed the bone marrow’s ability to upregulate erythropoiesis, resulting in anemia. It is important to mention that excessive eryptosis is also associated with various pathological conditions, including hypertension, dyslipidemia, diabetes mellitus, and hemolytic anemias [25].

Ca^2+^ signaling is perhaps the most important mechanism underlying PS translocation in RBCs [26]. Indeed, it was observed that ERN induced a substantial increase in cytosolic Ca^2+^ activity (Figure 4) which was seemingly independent of Ca^2+^ influx, as the exclusion of extracellular Ca^2+^ did not result in a significant decrease in cell death (Figure 7). Thus, Ca^2+^ channel activity does not appear to be necessary for ERN toxicity in RBCs, and the concurrent administration of Ca^2+^ channel blockers with ERN may not be of value. In response to an increase in intracellular Ca^2+^ activity, K^+^ channels mediate KCl efflux, membrane hyperpolarization, and water loss [27]. The consequence is significant cell shrinkage as a result of decreased cellular volume (Figure 6). Interestingly, we have also noted that ERN causes significant cell swelling, which is uncharacteristic of eryptotic cells. A possible explanation is that the loss of KCl was paralleled by Na^+^ entry leading to overhydration, as was observed in trifluoperazine-induced eryptosis [28]. Thus, the modulation of the Na^+^/K^+^-ATPase pump by ERN cannot be excluded, as it maintains cell volume by adjusting Na^+^ and K^+^ concentrations. This is likely given that ERN toxicity was blunted by isosmotic urea (Figure 7), which targets the Na^+^/K^+^-ATPase pump, KCl cotransport, and Na^+^-K^+^-2Cl^-^ transporters [29].

One of the canonical characteristics of eryptotic cells is oxidative stress, which contributes to life-threatening conditions such as cancer, diabetes mellitus, and hepatic failure [30]. Previous studies have demonstrated that elevated levels of ROS are associated with the overactivity of cation channels and the induction of eryptosis—a process dependent on Ca^2+^ mobilization [31]. The failure of erythrocytes to neutralize ROS results in a reduced oxygen-carrying capacity and promotes premature cellular aging and death [32] due to the oxidation of cellular components, most notably the plasma membrane, which may cause increased permeability and subsequent cellular swelling (Figure 6 and Figure 8) [33].

The utilization of small-molecule inhibitors has facilitated the elucidation of specific molecular mediators targeted by therapeutic interventions in erythrocytes. It was observed that the hemolytic activity of ERN was significantly reversed when CK1α or Rac1 GTPase were blocked. The involvement of CK1α in cellular viability is well-documented. It has a crucial role in differentiation, proliferation, and multiple forms of cell death, including autophagy, necroptosis, and pyroptosis [34]. Since CK1α has been identified as a key factor in Ca^2+^ entry through Cl^-^-responsive Ca^2+^ channels under conditions of energy depletion, oxidative stress, and osmotic shock [35], it is thus reasonable to assume the existence of an ATP/ROS-CK1α-Ca^2+^ molecular axis that drives erythrocyte death under certain stress conditions. Accordingly, AMPK, known to orchestrate a wide array of cell death modalities in nucleated cells and in erythrocytes [36,37], may be involved in the crosstalk between CK1α, Ca^2+^ mobilization, and metabolic exhaustion.

Rac GTPases are known to maintain the hexagonal architectural organization of the RBC cytoskeleton [38]. Previous studies have reported that Rac1 GTPase is essential for intracellular ROS formation by activating NADPH oxidases and nitric oxide synthase [39]. Inhibiting Rac1 GTPase activity with NSC23766 significantly reduced ERN-mediated hemolysis (Figure 7), suggesting that Rac1 GTPase participates in ERN toxicity to RBCs, most likely by promoting ROS formation [40]. Notably, oxysterols [41] and α-mangostin [42] have recently been reported as modulators of Rac1 GTPase in RBCs.

We have also identified other inhibitors of ERN toxicity, including sucrose, urea, and PEG 8000 (Figure 7). Although it still eludes us, the antihemolytic effect of sucrose is ascribed to multiple mechanisms. Sucrose may inhibit Cl^-^ exit, prevent colloid osmotic swelling by blocking water entry, or bind to ERN reducing its potency. Urea is efficiently transported across the membrane of RBCs through facilitated diffusion. The rapid transport of urea is responsible for preserving the osmotic stability and facilitates deformability across osmotic gradients, especially in the renal lumen [43]. Besides modulating channel activity, the protective effect of urea may in part be related to sphingomyelinase inhibition [29]. Of note, structural modifications may potentially reduce the toxicity of ERN to off-target tissue while preserving or enhancing its anticancer properties [44]. Interestingly, oncolytic viruses may also be exploited as vehicles of chemotherapeutic agents, which augments their anticancer potential to overcome chemoresistance [45].

## 4. Methods

### 4.1. Chemicals and Reagents

Solarbio Life Science (Beijing, China) supplied all chemicals and reagents unless stated otherwise. A stock solution of 10 mM of ERN (CAS number: 13463-28-0) was prepared by dissolving 10 mg in 1.68 mL of dimethyl sulfoxide (DMSO). The ringer solution contained 125 mM NaCl, 5 mM KCl, 1 mM MgSO_4_, 32 mM HEPES, 5 mM glucose, and 1 mM CaCl_2_, whereas the phosphate-buffered saline (PBS) used was composed of 137 mM NaCl, 2.7 mM KCl, 10 mM Na_2_HPO_4_, and 1.8 mM KH_2_PO_4_.

### 4.2. RBC Purification

The Ethics Committee of King Saud University Medical City approved the protocol for this study (E-23-7764). All participants provided informed consent according to the Declaration of Helsinki. Lithium heparin- or EDTA-anticoagulated blood samples were collected from 28 healthy volunteers (19 males and 9 females) aged 29–37 years with a BMI of ≤25 and normal CBC to isolate RBCs via centrifugation at 2500 RPM for 15 min at room temperature. Cells were washed several times in PBS or Ca^2+^-free Ringer solution and maintained at 4 °C for up to 24 h [46].

### 4.3. Experimental Design

RBCs at 5% hematocrit were exposed to 10–100 μM of ERN for 24 h at 37 °C. Where indicated, cells were exposed to 80 μM of ERN with and without CK1α inhibitor D4476 (20 µM), Rac1 GTPase inhibitor NSC23766 (100 µM), or ATP (500 μM). In certain experiments, Ca^2+^ was removed from the extracellular space, NaCl was replaced by 125 mM of KCl or 250 mM of sucrose, urea was added at 300 mM, or PEG 8000 was added at 10% *w*/*v* [47].

### 4.4. Hemolysis

The light absorbance of the supernatants obtained from the control and treated cells was measured at 405 nm using a LMPR-A14 microplate reader (Labtron Equipment Ltd., Surrey, UK). The cells suspended in ddH_2_O were used as a positive control (i.e., 100% hemolysis) to derive percent hemolysis as follows:

$$Hemolysis\left(\%\right)=\frac{OD\;blank-OD\;unknown}{OD\;blank-OD\;positivecontrol}\times 100$$

### 4.5. Potassium

Extracellular K^+^ in the supernatants of the control and treated cells was quantified using Solarbio’s Blood Potassium Content Assay Kit (catalog number: BC2775). In this assay, sodium tetraphenylborate combines with K^+^ in the sample to form a white K^+^ tetraphenylborate precipitate detected at 520 nm [48].

### 4.6. Lactate Dehydrogenase (LDH)

LDH activity was measured using Solarbio’s LDH Activity Assay Kit (catalog number: BC0685). In alkaline conditions, LDH converts NAD^+^ and lactic acid into NADH and pyruvate, which subsequently interacts with 2,4 dinitrophenylhydrazine to produce pyruvate dinitrobenzene (λ_max_ = 450 nm) [49]. One unit of enzyme activity is the amount of enzyme required to generate 1 nM of pyruvate per min per mL supernatant.

### 4.7. Aspartate Aminotransferase (AST)

AST activity was determined using Solarbio’s AST Activity Assay Kit (catalog number: BC1565) based on the Reitman–Frankel colorimetric method [50]. In the reaction mixture, AST catalyzes the transfer of an amino group from aspartate to α-ketoglutarate, generating glutamate and oxaloacetate, which is decarboxylated by oxaloacetate decarboxylase to pyruvate. The latter was reacted with 2,4-dinitrophenylhydrazine in an alkaline solution to produce brown-red 2,4-dinitrophenylhydrazone detected at 505 nm. One unit of enzyme activity is the amount of enzyme necessary to produce 1 μM of pyruvate per min per mL supernatant.

### 4.8. Creatine Kinase (CK)

A CK Activity Assay Kit (Solarbio; catalog number: BC1145) was used to measure CK activity. In a series of reactions, CK phosphorylates ADP to ATP, which is added to glucose by hexokinase to form glucose-6-phosphate. Glucose-6-phosphate dehydrogenase then converted glucose-6-phosphate to gluconate-6-phosphate, while NADP^+^ was reduced to NADPH, whose generation can be monitored at 340 nm [51]. One unit of activity is the amount of CK required to generate 1 nM of NADPH at 37 °C and pH 7.0 per min per mL supernatant.

### 4.9. Extracellular Acidity

The pH of the supernatants of the control and treated RBCs was measured using the EXIAS e|1 analyzer (EXIAS Medical GmbH, Graz, Austria) [47].

### 4.10. Acetylcholine Esterase (AChE)

Solarbio’s AChE Activity Assay Kit (catalog number: BC2025), based on the Ellman’s method, was used to measure AChE activity. AChE hydrolyzes acetylthiocholine into thiocholine, which produces 5-mercaptonitrobenzoic acid (λ_max_ = 412 nm) from 2-nitrobenzoic acid. One unit of activity is defined as the amount of AChE that generates 1 nM of 5-mercaptonitrobenzoic acid per min per mL hemolysate [52].

### 4.11. Membrane Asymmetry and Cellular Morphology

The control and experimental cells were stained with 1% annexin-V-FITC for 10 min at RT, and a total of 10,000 cells were analyzed using a Northern Lights flow cytometer (Cytek Biosciences, Fremont, CA, USA). FITC was stimulated using a blue laser at 488 nm, and the emitted green light was captured at 520 nm.

Cell size and granularity were determined from forward scatter (FSC) and side scatter (SSC) signals, respectively [53].

Samples were prepared for scanning electron microscopy (SEM) as previously detailed [18]. Briefly, the control and experimental cells (100 μM) were fixed in glutraldehyde, stained with osmium tetroxide, dried in ethanol, and examined using a JSM-7610F ultra-high resolution Schottky field emission scanning electron microscope (JEOL Co., Ltd., Akishima, Tokyo, Japan).

### 4.12. Intracellular Ca^2+^

Ca^2+^ in the control and treated cells was labeled with 2 µM of Fluo4/AM for 30 min at 37 °C and analyzed by flow cytometry at 512 nm [54].

### 4.13. Oxidative Stress

ROS were stained by 5 µM of 2′,7′-dichlorodihydrofluorescein diacetate (H_2_DCFDA) for 30 min at 37 °C and analyzed by flow cytometry at 520 nm [55].

### 4.14. Systemic Toxicity

A CBC was performed on whole blood diluted 1:2 with PBS following incubation for 24 h at 37 °C with and without 100 µM of ERN using a BC-6200 hematology analyzer (Mindray Medical International Limited, Shenzhen, China) [42].

### 4.15. Erythrocyte Sedimentation Rate (ESR)

The distance travelled by the RBCs in control and treated whole blood sedimented in Westergren tubes for 60 min was recorded [42].

### 4.16. Statistics

Flow cytometric outputs, expressed as arbitrary units (a.u.) or percentages, were analyzed using FlowJo^TM^ v10.7.2 (Becton, Dickinson and Company, Ashland, OR, USA), and GraphPad v9.5.1 (GraphPad Software, Inc., San Diego, CA, USA) was used to perform statistical analyses. Data are shown as means ± SEM of three independent experiments. Two groups were compared using Student’s *t*-test, while three or more were compared via a one-way ANOVA corrected by Dunnett’s post hoc test. Significance was set at a *p* value of <0.05.

## 5. Conclusions

In conclusion, this study reports that ERN elicits hemolysis and eryptosis characterized by a decrease in membrane asymmetry, Ca^2+^ mobilization, cell shrinkage and swelling, and oxidative injury. Moreover, CK1α and Rac1 GTPase have been identified as essential for the toxic effects of ERN in RBCs, which can be abrogated by isosmotic sucrose, urea, and PEG 8000. The further development and validation of ERN in anticancer therapy must be cautiously pursued.

## Data Availability

Data is contained within the article.
